# Phenotypic variation and diversity assessment in maize landraces

**DOI:** 10.1371/journal.pone.0355741

**Published:** 2026-08-14

**Authors:** Longxue Wei, Lianghai Guo, Dongbo Zhao, Jianjun Guo, Yirong Jin, Jiansheng Gao, Huini Cui, Liang Zhang, Peng Liu, Zhihui Guo

**Affiliations:** Dezhou Agricultural Science Research Institute, Dezhou, Shandong, China; KGUT: Graduate University of Advanced Technology, IRAN, ISLAMIC REPUBLIC OF

## Abstract

Maize landraces are invaluable reservoirs of genetic diversity for crop improvement, yet systematic evaluation of their quantitative traits remains insufficient. In this study, 119 maize landraces were evaluated for nine quantitative traits related to yield and tassel morphology. Genetic diversity indices, principal component analysis(PCA), cluster analysis, and stepwise regression were applied to characterize phenotypic variation and identify core evaluation indicators. The landraces exhibited substantial genetic variation, with coefficients of variation ranging from 20.73% to 60.96% and Shannon-Winner index from 0.88 to 0.98. PCA extracted four principal components explaining 68.81% of the total variation, with the first component (24.17%) primarily driven by the number of secondary tassel branches. Based on comprehensive D values (0.033–0.960), ten superior landraces were identified, including Z102, Z7, and Z89. Cluster analysis classified the 119 landraces into three groups differing in tassel morphology, grain characteristics, and agronomic performance. Stepwise regression further identified four key indicators—number of secondary tassel branches, angle between primary tassel branches and main axis, tassel diameter, and number of spike rows—as the most informative traits for breeding selection. These results provide a practical framework for the efficient exploitation of maize landrace resources and the identification of elite breeding materials.

## Introduction

Maize, cultivated on approximately 200 million hectares worldwide, is a critical crop for global food security [1]. The foundation of maize genetic improvement lies in the effective utilization of diverse germplasm resources, which provide the raw materials necessary for breeding innovations [2]. Among these resources, maize landraces hold particular importance due to their strong adaptability to local environments, stress resistance, and rich genetic variation. Historically, they have not only contributed directly to maize production in regions such as China but also established the genetic basis for modern hybrid breeding [3]. Therefore, a comprehensive and systematic study of these resources is a prerequisite and key to managing and utilizing them effectively. The current narrow genetic base of hybrid maize varieties underscores the urgency of systematically characterizing and utilizing maize landraces to broaden the genetic diversity available for breeding programs [4].

Maize landraces represent the most diverse genetic resource within maize germplasm, making them indispensable for overcoming challenges such as germplasm bottlenecks, variety homogenization, and reduced breeding efficiency [5–8]. Global studies have documented the extensive genetic diversity and population structure of maize landraces across diverse agro-ecological zones, including Peru [9], Mexico [10], Tunisia [11], northern Argentina [12], Europe [13]), and central China [14]. These findings highlight the valuable variation present in local maize germplasm and reinforce the need for targeted evaluation. Phenotypic traits, as the observable morphological and agronomic characteristics, provide a direct measure of germplasm diversity shaped by the interaction of genetics and environment. Evaluating phenotypic diversity is fundamental for understanding germplasm resources, mining beneficial genes, and guiding the development of new varieties [15,16]. For instance, research on Chinese maize landraces revealed high phenotypic diversity, especially in Southwest China, and identified favorable alleles such as TU1 linked to high yield potential [17,18]. Additionally, the rhizosphere microorganisms associated with maize landraces have been shown to promote plant growth and disease resistance, further emphasizing their breeding value [19].

Their research still has following limitations: Firstly, it is based on the molecular level population genetic analysis, without the support of the systematic phenotypic quantification data for the local variety resources, which leads to the disconnection of “genotype” and “phenotype”. Secondly, even if phenotypic identification is conducted, it is mostly limited to the broad trait description, without the in-depth analysis on the correlation between the key traits, nor the establishment of the efficient quantitative evaluation criteria for the selection of excellent germplasm. For Chinese maize landraces, phenotypic variation has been reported, but there is rare attention to tassel configuration, an inflorescence trait controlled by high heritability, and resource classification and utilization potential evaluation based on statistical models.

This study focuses on the phenotypic traits of local maize landraces, employing statistical analyses to assess their diversity and comprehensively evaluate their performance. The objectives are to develop efficient evaluation criteria, explore trait correlations, and identify superior germplasm resources. These efforts aim to provide a robust foundation for the conservation, utilization, and breeding of maize landraces, thereby accelerating the innovative application of these valuable genetic resources.

## Materials and methods

### Test materials

In this study, a total of 119 maize landraces were collected, hailing from the Huang-Huai production regions, including Shandong, Shanxi, Shanxi, Anhui, and Henan provinces. These varieties were obtained from the National Maize Germplasm Resources Bank and supplied by the Institute of Crop Sciences at the Chinese Academy of Agricultural Sciences (S1 Table). Comprehensive details regarding these varieties are presented in Table 1.

**Table 1:** Codes, D values of comprehensive evaluation and ranking of 119 maize landraces.

Code	D -value	Ranking	Code	D -value	Ranking	Code	D-value	Ranking	Code	D -value	Ranking
Z1	0.533	38	Z31	0.477	50	Z61	0.348	81	Z91	0.340	83
Z2	0.404	65	Z32	0.179	111	Z62	0.545	35	Z92	0.402	66
Z3	0.355	78	Z33	0.461	56	Z63	0.499	47	Z93	0.629	14
Z4	0.148	114	Z34	0.256	100	Z64	0.319	89	Z94	0.720	7
Z5	0.429	62	Z35	0.475	51	Z65	0.227	105	Z95	0.293	93
Z6	0.365	75	Z36	0.112	116	Z66	0.582	24	Z96	0.399	68
Z7	0.809	2	Z37	0.050	118	Z67	0.572	27	Z97	0.468	53
Z8	0.164	113	Z38	0.209	108	Z68	0.364	76	Z98	0.741	6
Z9	0.437	59	Z39	0.551	33	Z69	0.677	9	Z99	0.401	67
Z10	0.583	23	Z40	0.335	85	Z70	0.174	112	Z100	0.516	40
Z11	0.593	18	Z41	0.332	86	Z71	0.291	96	Z101	0.541	37
Z12	0.650	12	Z42	0.348	80	Z72	0.570	28	Z102	0.960	1
Z13	0.504	45	Z43	0.346	82	Z73	0.462	55	Z103	0.482	49
Z14	0.293	94	Z44	0.254	102	Z74	0.776	4	Z104	0.581	25
Z15	0.453	57	Z45	0.394	70	Z75	0.352	79	Z105	0.508	43
Z16	0.512	42	Z46	0.209	107	Z76	0.270	98	Z106	0.603	17
Z17	0.338	84	Z47	0.244	103	Z77	0.512	41	Z107	0.294	92
Z18	0.272	97	Z48	0.470	52	Z78	0.504	44	Z108	0.627	15
Z19	0.555	31	Z49	0.487	48	Z79	0.436	60	Z109	0.500	46
Z20	0.083	117	Z50	0.466	54	Z80	0.189	109	Z110	0.709	8
Z21	0.239	104	Z51	0.377	74	Z81	0.357	77	Z111	0.592	19
Z22	0.291	95	Z52	0.436	61	Z82	0.417	63	Z112	0.578	26
Z23	0.437	58	Z53	0.660	11	Z83	0.381	73	Z113	0.751	5
Z24	0.547	34	Z54	0.409	64	Z84	0.527	39	Z114	0.615	16
Z25	0.585	22	Z55	0.569	29	Z85	0.553	32	Z115	0.636	13
Z26	0.588	20	Z56	0.212	106	Z86	0.566	30	Z116	0.670	10
Z27	0.544	36	Z57	0.387	71	Z87	0.255	101	Z117	0.331	87
Z28	0.326	88	Z58	0.305	91	Z88	0.144	115	Z118	0.397	69
Z29	0.307	90	Z59	0.262	99	Z89	0.790	3	Z119	0.033	119
Z30	0.381	72	Z60	0.182	110	Z90	0.587	21			

### Test method

#### Overview of test site.

The experiment was conducted in the modern agricultural science and Technology Industrial Park in Huangheya Town, Dezhou City, Shandong Province in 2024. Sown on June 13, the average sunshine duration of maize growth period was about 7.4 hours, the average temperature was about 24.2 °C, and the total precipitation was about 397 mm. It was harvested on October 10 of the same year. There was no abnormal phenomenon in the average temperature of maize growth period. The 119 maize landraces used in this experiment had consistent environmental variables at the test site.

#### Test design.

The trial was designed as a completely randomized block trial with three replicates. Each resource was planted in 3 rows, with a length of 3 m, a row spacing of 0.6 m, a plant spacing of 22.2 cm, and a plot planting density of 75000 plants hm^-2^. The experiment site has sufficient light, the cultivation management measures and water and fertilizer conditions were the same as the local field management measures, and the pest control was carried out according to the conventional conditions.

#### 9 quantitative traits.

Number of spike rows (SRN): the number of rows of kernels in the middle of the spike at grain maturity;

Number of grains per row (GPR): number of grains per row of spike at grain maturity;

1000-grain weight (TGW): the weight of 1000 seeds at grain maturity;

Tassel stalk length (TSL): tassel and stem node to tassel base branch point;

Tassel principal axis length (TPAL): the length from the branch point of tassel base to the top, measured with a ruler, expressed in cm;

Diameter of tassel stalk (TSD): measured with vernier caliper, expressed in cm;

Number of tassel branches(TBN): investigate the number of branches disposed by tassel principal axis at silking stage;

Secondary branch number of tassel(TSBN): the number of re branches at the primary branch of tassel was investigated at silking stage;

Angle between tassel primary branch and tassel principal axis(AFBMA): investigate the angle between tassel primary branch and tassel principal axis at silking stage, and measure it with protractor in degrees.

### Data analysis

#### Membership function analysis.


$$\begin{array}{c} u\left(xi\right)=\left(xi-ximin\right)/\left(ximax-ximin\right)\left(i=1,2,3,...,n\right) \end{array}$$ [1]

In the formula, u (X_i_) is the membership function value of the ith character of each material, X_i_ is the value of the ith character of each material, and x_imax_ and X_imin_ are the maximum and minimum values of the ith character of all tested materials respectively.

#### Genetic diversity index.

The relative frequency of each grade of each trait was obtained by the membership function, and then the Shannon Wiener diversity index (H ′) was used to evaluate the genetic diversity.


$$\begin{array}{c} H^{\prime }=-\sum_{i=1}^{n}Pi\text{\textbackslash{}hspace\{0.17em}\;\}\times \text{\textbackslash{}hspace\{0.17em}\;\}lnPi\left(i=1,2,3,...,n\right) \end{array}$$ [2]

In the formula, *Pi* is the percentage of the number of materials in grade i of a certain character in the total number of materials, ln is the natural logarithm [20].

Principal component analysis weight coefficient of principal component:


$$\begin{array}{c} P=Ci/\sum_{n=1}^{n}Ci\text{\textbackslash{}hspace\{0.17em}\;\}(i\text{\textbackslash{}hspace\{0.17em}\}=\text{\textbackslash{}hspace\{0.17em}\}1,\text{\textbackslash{}hspace\{0.17em}\;\}2,\text{\textbackslash{}hspace\{0.17em}\;\}3,\;\ldots ,n) \end{array}$$ [3]

Where *Ci* is the contribution rate of the ith principal component.

##### Comprehensive score of maize landraces in each:


$$\begin{array}{c} W\text{i}=\text{\textbackslash{}hspace\{0.17em}\}\sum_{i=1}^{n}PiZi\text{\textbackslash{}hspace\{0.17em}\;\}(i\text{\textbackslash{}hspace\{0.17em}\}=\text{\textbackslash{}hspace\{0.17em}\;\}1,\text{\textbackslash{}hspace\{0.17em}\;\}2,\text{\textbackslash{}hspace\{0.17em}\;\}3,\ldots ,n) \end{array}$$ [4]

Where *Pi* is the weight coefficient of the ith principal component, and *Zi* is the score of the ith principal component of the corresponding maize germplasm.

##### Comprehensive evaluation.

The formula for calculating the comprehensive score:


$$\begin{array}{c} D=\sum_{I}=L\text{n}\left(D\left(X\text{i}\right)\times W_{\text{i}}\right) \end{array}$$ [5]

##### Stepwise regression analysis.

Using the comprehensive score D value as the dependent variable and 9 trait indicators as independent variables, a stepwise regression analysis was conducted to screen key indicators.

#### Data processing

Excel 2018 was used to calculate the mean and standard deviation of each trait. The quantitative traits were measured repeatedly for three times, and the average of the three measured values was taken as the result SPSS 26.0 software (IBM company, New York, USA) was used to calculate the coefficient of variation and analyze the frequency distribution of different character types. The Shannon-Wiener index was calculated using Excel 2018. Circular visualization, including enhancements and implementations, was performed in the Microbioinformatics cloud platform (www.bioinformatics.com.cn), which also facilitated z-score transformation and Euclidean distance-based complete clustering. Origin 2021 was used to draw the grouped violin diagram, IBM SPSS statistics 26 conducted analysis of variance (one-way ANOVA, Duncan method), correlation analysis (Pearson, Perform statistical analysis on the mean and standard deviation, conduct a two-tailed test of significance, and exclude paired cases. *P* = 0.05, *P* = 0.01), stepwise regression analysis (a linear regression approach using the stepwise method, where normal distribution is confirmed by the Durbin-Watson test; default settings include: a significance level of 0.05 for introducing variables, ANOVA with an F-to-enter value of <0.05 and an F-to-remove value of >0.10, and output of tolerance and VIF for collinearity diagnosis) and constructed the optimal regression equation, and conducted principal component analysis, calculated the scores of each principal component and the D value of the comprehensive score for comprehensive evaluation, and screened excellent germplasm.

### Results and analysis

#### Phenotypic diversity analysis of maize germplasm resources

Descriptive statistical analysis was carried out on 9 phenotypic quantitative traits of 119 maize landraces (S2 File). The coefficient of variation of 9 phenotypic traits ranged from 20.73% to 60.96%, and the genetic diversity index calculated across traits ranged from 0.88 to 0.98. Among the 9 quantitative traits of the tested materials, 1000 grain weight, the angle between the first branch of tassel and the main axis, etc., had a larger variation range; The coefficient of variation of the number of secondary branches of tassels was the largest (60.96%), followed by the coefficient of variation of the angle between the first branches and the main axis of tassels (52.72%); The average coefficient of variation for the seven other quantitative traits was 25.74%. The phenotypic diversity index, ranked from highest to lowest, was: 1000-grain weight (0.98), number of grains per row (0.97), number of secondary tassel branches (0.94), tassel stalk diameter (0.93), tassel stalk length (0.92), tassel principal axis length (0.91), tassel branches and number of rows per spike (both 0.89), and angle between the first tassel branches and principal axis (0.88).The results showed that the genetic variation of phenotypic traits of the 119 maize landraces were rich, and the genetic diversity was high(Table 2).

**Table 2:** Phenotypic diversity and variation analysis of 9 quantitative traits in tested materials.

Traits	Variation range	Mean±SD	median	Kurtosis	Skewness	coefficient of variation %	Shannon-wiener index
**SRN**	8.00-26.00	13.27 ± 2.98	12.00	2.58	1.11	22.46%	0.89
**GPR**	15.00-46.00	30.67 ± 7.59	30.00	−0.99	0.05	24.76%	0.97
**TGW**	122.37-450.10	264.42 ± 70.23	259.43	−0.77	0.15	26.56%	0.98
**TSL**	6.60-40.20	19.05 ± 5.48	19.00	0.88	0.42	28.75%	0.92
**TPAL**	17.30-59.60	36.74 ± 7.62	36.90	−0.20	0.15	20.73%	0.91
**TSD**	0.40-1.15	0.67 ± 0.15	0.65	−0.02	0.28	21.93%	0.93
**TBN**	2.00-39.00	18.32 ± 6.41	18.00	0.23	0.12	34.96%	0.89
**TSBN**	0.00-17.00	5.75 ± 3.51	5.00	−0.46	0.41	60.96%	0.94
**AFBMA**	8.00-118.00	38.59 ± 20.35	35.00	0.09	0.72	52.72%	0.88

Note: SRN:spike row number; GPR:grains per row; TGW:1000 grain weight; TSL:tassel stalk length; TPAL:tassel principal axis length; TSD:tassel stalk diameter; TBN:tassel branch number; TSBN:tassel secondary branch number; AFBMA:The angle between the first branch of tassel and the main axis of tassel; Same as below.

#### Correlation analysis

In order to understand the correlation between the phenotypic traits of the tested resources, the correlation analysis was carried out on the 9 traits, and the Pearson correlation coefficient was used to express the strength of the correlation. There were extensive correlations among 21 pairs of traits among 9 phenotypic traits, reaching significant and extremely significant correlation levels. Specific analysis showed that:

The correlation coefficients between the number of rows per spike and the number of grains per row, the number of rows per spike and 1000-grain weight, and the number of rows per spike and tassel stalk diameter were −0.16, −0.40, and 0.16, respectively, all significant at the 0.01 level (S3 Table). The correlations between grains per row and 1000 grain weight, grains per row and:tassel principal axis length; grains per row and tassel stalk diameter; as well as grains per row and tassel secondary, all reached significance at the 0.01 level. The results showed that there were significant positive correlations:between 1000 grain weight and tassel stalk length, between 1000 grain weight and tassel principal axis length, between 1000 grain weight and tassel secondary branch number; There was a significant negative correlation between tassel stalk length and tassel branch number (*p* < 0.01, −1 < r < 0). The correlations between tassel principal axis length and tassel stalk diameter, tassel principal axis length and:tassel secondary branch number, all reached significance at the 0.01 level. The correlations between tassel stalk diameter and tassel stalk diameter,tassel stalk diameter and:tassel secondary branch number, all reached significance at the 0.01 level.The correlations between tassel branch number and tassel stalk diametertassel branch number tassel branch number and the angle between the first branch of tassel and the main axis of tassel, all reached significance at the 0.01 level. In addition to the number of tassel branches, It can be seen that there is a certain correlation between the three factors of maize yield (row number per spike, grain number per row, 1000 grain weight) and the tassel traits of maize, and the tassel of maize plays an important role in the formation of yield(Table 3).

**Table 3:** Pearson correlation analysis of 9 quantitative traits in tested materials.

Treats	SRN	GPR	TGW	TSL	TPAL	TSD	TBN	TSBN	AFBMA
**SRN**	1								
**GPR**	−0.16**	1							
**TGW**	−0.40**	0.28**	1						
**TSL**	0.04	0.21**	0.19**	1					
**TPAL**	−0.03	0.26**	0.15**	0.05	1				
**TSD**	0.16**	0.30**	0.11*	0.02	0.32**	1			
**TBN**	−0.01	−0.07	0.08	−0.24**	0.11*	0.21**	1		
**TSBN**	−0.02	0.14**	0.21**	−0.04	0.27**	0.44**	0.49**	1	
**AFBMA**	0.11*	−0.01	−0.05	0.09	0.04	−0.06	0.29**	0.01	1

Note: SRN: spike row number; GPR: grains per row; TGW: 1000 grain weight; TSL: tassel stalk length; TPAL: tassel principal axis length; TSD: tassel stalk diameter; TBN: tassel branch number; TSBN: tassel secondary branch number; AFBMA: The angle between the first branch of tassel and the main axis of tassel.

*: p < 0.05, **: p < 0.01

#### Principal component analysis

By conducting principal component analysis (PCA) on nine phenotypic traits, we extracted four principal component factors. The eigenvalues of the four principal components were 2.18, 1.63, 1.27 and 1.12, which were greater than 1, and the cumulative variance interpretation rate was 68.81%, which could explain 68.81% of the information of the nine investigated quantitative traits (S4 File). The contribution rate of the first principal component was the largest (24.17%), which was closely related to the number of secondary branches of tassel. The contribution rate of the second principal component was 18.07%, which was closely related to the number of tassel branches. The contribution rates of the third principal component and the fourth principal component were 14.07% and 12.49%, respectively, which were closely related to the number of rows per spike and the angle between the first branch and the main axis of tassel, respectively. It can be seen that the original nine trait indicators can be transformed into four independent comprehensive indicators through principal component analysis, which can be used to build a linear model of four principal components and carry out comprehensive evaluation and analysis (Table 4).

**Table 4:** Eigen values and eigenvectors of first four principal components.

Trait	Principal component			
**F1**	**F2**	**F3**	**F4**
**SRN**	−0.17	0.51	0.70	−0.01
**GPR**	0.52	−0.48	0.25	0.03
**TGW**	0.49	−0.53	−0.37	0.16
**TSL**	0.08	−0.50	0.47	0.47
**TPAL**	0.59	−0.05	0.24	−0.05
**TSD**	0.68	0.16	0.38	−0.29
**TBN**	0.50	0.61	−0.37	0.21
**TSBN**	0.75	0.30	−0.11	−0.10
**AFBMA**	0.08	0.33	0.04	0.86
**Eigenvalue**	2.18	1.63	1.27	1.12
**Contribution percentage (%)**	24.17	18.07	14.07	12.49
**Cumulative contribution percentage (%)**	24.17	42.25	56.31	68.81

Note: SRN: spike row number; GPR: grains per row; TGW: 1000 grain weight; TSL: tassel stalk length; TPAL: tassel principal axis length; TSD: tassel stalk diameter; TBN: tassel branch number; TSBN: tassel secondary branch number; AFBMA: The angle between the first branch of tassel and the main axis of tassel; The same below.

#### Comprehensive evaluation

The principal component score was used for comprehensive evaluation, and the “linear combination coefficient matrix” was used to establish the relationship equation between the principal component and the research item (the relationship expression is established based on the standardized data), as follows:


$$\begin{array}{c} W1=-0.113X1+0.351X2+0.332X3+0.055X4+0.402X5+0.462X6+0.340X7+0.507X8+0.054X9 \end{array}$$

$$\begin{array}{c} W2=0.402X1-0.379X2-0.412X3-0.395X4-0.043X5+0.124X6+0.477X7+0.237X8+0.261X9 \end{array}$$

$$\begin{array}{c} W3=0.623X1+0.221X2-0.332X3+0.413X4+0.215X5+0.336X6-0.333X7-0.100X8+0.036X9 \end{array}$$

$$\begin{array}{c} W4=-0.012X1+0.027X2+0.151X3+0.439X4-0.049X5-0.275X6+0.198X7-0.098X8+0.810X9 \end{array}$$

In the formula, X1 ~ X9 are respectively: X1:spike row number; X2:grains per row; X3:1000 grain weight; X4:tassel stalk length; X5:tassel principal axis length; X6:tassel stalk diameter; X7:tassel branch number; X8:tassel secondary branch number; X9:The angle between the first branch of tassel and the main axis of tassel.

The scores of the four principal components are normalized by using the fuzzy membership function to calculate the comprehensive score D value: D value = 0.351W1+0.263W2 + 0.204W3+0.182W4.

The D values of 119 maize landraces ranged from 0.033 to 0.960 (Table 1), and the top ten maize landraces with D values were Z102, Z102 and Z102, respectively Z7, Z89, Z74, Z113, Z98, Z94, Z110, Z69, Z116.

#### Cluster analysis and characteristics of various clusters

**Fig 1:**
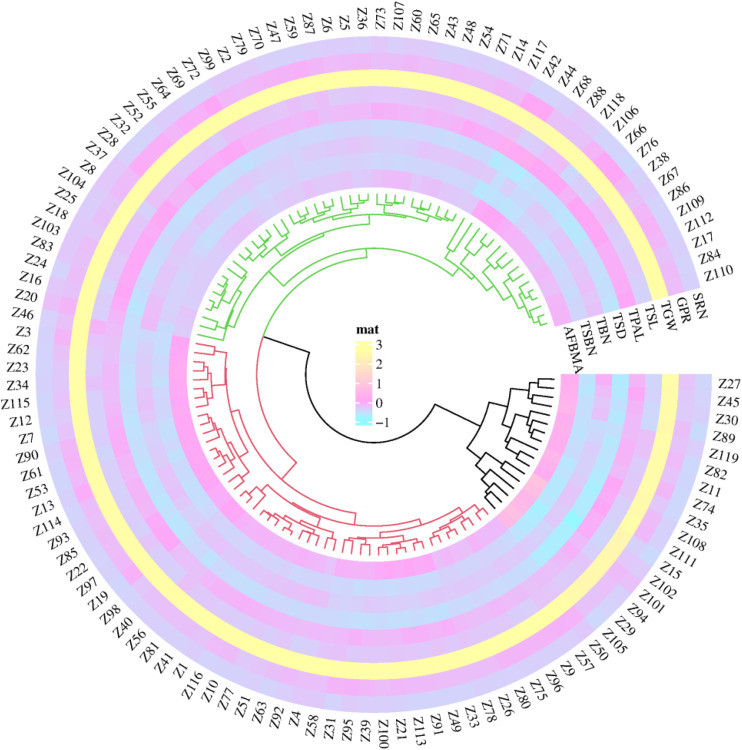
Cluster analysis of comprehensive scores of 119 maize landraces. Note:SRN: spike row number; GPR: grains per row; TGW: 1000 grain weight; TSL: tassel stalk length; TPAL: tassel principal axis length; TSD: tassel stalk diameter; TBN: tassel branch number; TSBN: tassel secondary branch number; AFBMA: The angle between the first branch of tassel and the main axis of tassel; Same as bellow. Cluster tree color: Black represents Group Ⅰ; Red represents Group Ⅱ; Green represents Group Ⅲ.

Based on 9 quantitative traits for systematic clustering analysis, 119 maize landraces were divided into 3 categories (S5 File). Cluster Ⅰ includes 17 maize landraces, accounting for 14.29% of all maize landraces, with a D value range of 0.033–0.960; Cluster Ⅱ includes 49 maize landraces, accounting for 41.18% of all maize landraces, with a D value range of 0.148–0.751; Cluster Ⅲ includes 53 maize landraces, accounting for 44.54% of all maize landraces, with a D value range of 0.050–0.677 (Fig 1).

**Fig 2:**
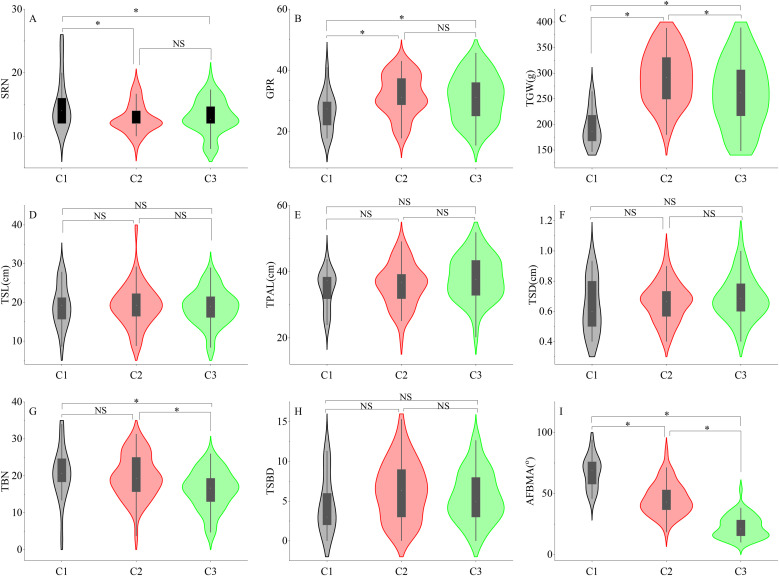
Comparison of the phenotypic differences of three cluster groups of maize landraces. Note: A: spike row number; B: grains per row; C: 1000 grain weight; D: tassel stalk length; E: tassel principal axis length; F: tassel stalk diameter; G: tassel branch number; H: tassel secondary branch number; I: The angle between the first branch of tassel and the main axis of tassel. NS: The difference is not significant; *: There is a significant difference at the 0.05 level. C1: Cluster Ⅰ (n = 17); C2: Cluster Ⅱ (n = 49); C3: Cluster Ⅲ (n = 53).

Further comparison of the three yield factors and tassel correlation traits among the three clusters showed that there were no significant differences among the three clusters for the four traits of tassel stalk length, tassel principal axis length, tassel stalk diameter, and tassel secondary branch number. However, there were significant differences among the three clusters for the two traits of thousand grain weight and the angle between the first branch of tassel and the main axis of tassel (Fig 2).

There were significant differences (*p* < 0.05) in the three essential traits of yield between cluster I and other clusters, with lower number of grains per row and thousand grain weight compared to other clusters. The angle between the first branch of tassel and the main axis of tassel of cluster I was higher than that of other clusters, and the difference between them was significant. The number of spike rows was 15.29, the number of grains per row was 26.47, the thousand grain weight was 193.45 g, the length of tassel stalk was 19.21 cm, the length of tassel principal axis was 34.70 cm, the diameter of tassel stalk was 0.63 cm, the number of tassel branches was 20.67, the number of secondary branches per tassel was 4.69, and the angle between the first branch of tassel and the main axis was 67.51 °. The main characteristics of this cluster were slightly more rows per spike, but slightly lower grain number and thousand grain weight per row. The angle between the first branch and the main axis of the tassel was large, and the overall performance was long bald tips, low grain density, and flat branches of the tassel.

Compared with the other two clusters, Cluster II has the fewest number of rows per spike, with higher row grain count, thousand grain weight, tassel stalk length, and secondary branch count. However, only the thousand grain weight and the angle between the first branch and the main axis of the tassel show significant differences compared to other clusters. The main characteristics of this clusters were slightly higher number of grains per row, slightly higher thousand grain weight, overall slender spikes, slightly longer tassel stalks, slightly more secondary branches of per tassel, and moderate other traits.

Comparison between Cluster I and Cluster II showed that Cluster III has the shortest tassel stem length, the fewest number of tassel branches, the smallest angle between the first branch and the main axis of tassel, the longest principal axis length, and the largest tassel stalk diameter. The main characteristics of this cluster were that the tassel stalk was thick and short, the main axis is slightly long, the tassel branches were few and clustered, and the other traits were moderate.

#### Stepwise regression analysis

To screen the evaluation and identification indicators for the three factors of yield and tassel traits in maize landraces, 9 trait values were used as independent variables, and the comprehensive evaluation D value was used as the dependent variable. Through stepwise regression analysis, the optimal regression equation was constructed as follows:

D = −0.402 + 0.024 X8 + 0.004 X9 + 0.515 X6 + 0.014 X1, In the formula, X8, X9, X6, and X1 respectively represent the number of secondary branches of the tassel, the angle between the first branches and the main axis of of the tassel, the diameter of the tassel stalk, and the number of spikes. The coefficient of determination for this equation was R^2^ = 0.952, *P* < 0.0001, This indicated that the constructed regression equation has a highly significant linear relationship. Therefore, the number of secondary branches of the tassel, the angle between the first branches and the main axis of the tassel, the diameter of the tassel stalk, and the number of spike rows could be used as evaluation indicators for the excellent traits of maize landraces. these traits may be utilized for the selection of traits in maize breeding.

### Discussion

Phenotypic traits encompass both qualitative and quantitative characteristics and remain one of the most direct and foundational approaches for assessing plant genetic diversity [21]. In diversity analyses, the coefficient of variation (CV) primarily reflects the magnitude of phenotypic variation within a trait—essentially the relative spread of trait values across accessions [22–23]. By contrast, the Shannon diversity index (H′) captures the distribution pattern of phenotypic classes or categories, integrating both richness (the number of distinct phenotypic states) and evenness (their relative abundance) [24]. It is therefore important to distinguish these metrics conceptually: CV emphasizes the intensity of variation, whereas H′ emphasizes the structural complexity of diversity.

Crop improvement fundamentally depends on the presence and accessibility of genetic diversity [25]. As the bridge between genotype and phenotype, phenotypic traits provide essential information for germplasm evaluation, utilization, and the dissection of complex agronomic traits [26]. In this study, nine quantitative phenotypic traits were measured across 119 maize landraces. The coefficients of variation ranged from 20.73% to 60.96%, while genetic diversity indices (H′) ranged from 0.88 to 0.98. These values indicate not only substantial phenotypic variation but also relatively high evenness in trait-state distribution, suggesting that the studied landraces harbor considerable genetic diversity with potential utility in breeding.

These findings align with previous reports emphasizing the richness of genetic variation in traditional maize germplasm. Using combined phenotypic and SSR data, Wei et al. [27] demonstrated that 102 local maize varieties from southwestern China possess abundant genetic variability and unique alleles. Similarly, Nelimor et al. [28] and Aleksandar et al. [29] highlighted the high degree of phenotypic and genetic variation characteristic of farmer-managed maize landraces. The present results are consistent with these conclusions, reinforcing the view that landraces represent reservoirs of adaptive and functional diversity. Beyond general diversity, several studies have pointed to their practical breeding value: Nelimor et al. [30] suggested that maize landraces can serve as sources of drought tolerance [31]; Gaikpa et al. [32] showed that high genetic variation in European maize cultivars could be effectively exploited through integrated genomic tools to improve resistance to Fusarium head blight; and Mayer et al. [33] reported that landrace-derived haplotypes often outperform those from modern breeding lines in key agronomic traits.

Among the nine quantitative traits analyzed here, three were yield-related (rows per ear, grains per row, thousand-grain weight), and six described tassel architecture. Traits exhibiting particularly pronounced variation included thousand-grain weight and the angle between the first tassel branch and the main axis. Given that yield-related traits directly influence overall productivity [34–35], such variation implies tangible potential for yield improvement. Furthermore, maize flowering traits—tasseling, silking, and pollen shedding—represent a critical window for grain set and are highly sensitive to high-temperature stress during reproductive development [36–38]. The observed phenotypic variation among the 119 landraces may thus encompass valuable alleles or trait combinations relevant to both yield stability and climate resilience.

Yield in maize is determined by a suite of interrelated traits, including effective spike number, rows per ear, grains per row, and thousand-grain weight. While spike traits generally show positive associations with yield [39], the strength and direction of these relationships can vary depending on genetic background and environmental conditions [40]. In this study, pairwise correlation analysis among nine phenotypic traits revealed 21 significant associations, many reaching p < 0.05. Yield components—rows per ear, grains per row, and thousand-grain weight—were strongly intercorrelated; however, the relationships were not uniformly positive. Thousand-grain weight was positively associated with grains per row but negatively correlated with rows per ear. Such counterintuitive patterns likely reflect compensation effects, whereby increased sink capacity (more rows) limits resource allocation per grain, reducing individual grain mass under fixed genetic or environmental constraints. These findings partially contrast with Pallabi et al. [41], who reported uniformly positive correlations between yield and rows per ear, grains per spike, and thousand-grain weight. The discrepancy may stem from differences in germplasm structure, population size, or growing environments, underscoring the context-dependency of trait–yield relationships in maize. Tassel architecture represents another key determinant of yield potential. As the primary male reproductive organ, the tassel influences plant resource allocation and photosynthetic efficiency [42]. In this study, the number of tassel branches was negatively correlated with both rows per ear and grains per row, aligning with previous evidence that excessive tassel branching reduces yield [43]. Biologically, this trade-off can be explained by Lambert et al. [44], who demonstrated that tassels develop apically and precede ear formation. Excessive branching increases vegetative investment, intensifying nutrient competition with developing ears and generating surplus pollen that shades leaves, thereby impairing photosynthesis. Consequently, tassel branch number emerges as a pivotal trait linking plant architecture with yield efficiency.

Maize tassel branch number is quantitatively inherited, governed by multiple genetic loci with high heritability (>80%) and primarily additive and dominant gene action, showing little epistasis [45–46]. This genetic architecture makes it amenable to selection, particularly in breeding programs targeting compact plant types and improved resource-use efficiency. Collectively, these results highlight that maize yield is shaped not merely by the magnitude of individual traits but by their balance and coordination. Negative correlations among yield components emphasize the importance of optimizing trait combinations rather than maximizing single traits. Compared with earlier studies, our findings reinforce the view that maize landraces maintain complex trait networks balancing reproductive allocation and resource efficiency. Leveraging this diversity could enable breeders to fine-tune yield architectures suited to both high-input systems and stress-prone environments.

Principal Component Analysis (PCA) was employed to reduce the dimensionality of the nine phenotypic traits, transforming them into a smaller set of uncorrelated synthetic variables that capture the majority of the phenotypic variation [47]. Four principal components (PCs) were extracted, explaining a cumulative variance of 68.81% among the 119 maize landraces. A deeper interpretation of the loadings reveals distinct biological themes. PC1 was heavily weighted by the number of tassel branches and secondary branches, representing vegetative allocation to the male inflorescence; this axis essentially contrasts excessive tassel proliferation against compactness. PC2, dominated by rows per ear and grains per row, represents yield sink capacity. PC3 and PC4 were defined by the tassel branch angle and thousand-grain weight, respectively, reflecting plant architecture and grain-filling efficiency. These components illustrate that phenotypic diversity in these landraces is structured around the trade-off between resource allocation to reproduction and vegetative growth. By integrating PCA scores with membership function analysis, we derived a comprehensive evaluation index (D-value, range: 0.033–0.960) to rank the germplasm. Landraces such as Z102, Z7, and Z89 emerged as superior resources. This multivariate approach refines traditional classification methods [48] and translates complex trait data into actionable breeding metrics. Crucially, since PC1 and PC2 capture traits known to be controlled by additive and dominant genes with high heritability [45–46], the identified elite materials provide a robust foundation for marker-assisted selection (MAS) and genomic selection (GS) [49].

Our findings align with previous reports of rich diversity in rural maize varieties [27–28] but extend them by highlighting the antagonistic relationship between tassel proliferation (PC1) and yield components (PC2). This supports the hypothesis that excessive tassel growth intensifies competition for resources, thereby limiting yield potential [44]. Compared to studies focusing solely on genetic distances, our phenotypic component analysis offers a clearer target for breeders: selecting for reduced tassel branching while maintaining high sink capacity. While the integration of phenomics [50] and genome-wide association studies (GWAS) [51] promises further advances, this study provides a practical framework for utilizing landraces. However, we acknowledge that the performance of these landraces is subject to genotype-by-environment interactions [49,52]. Future work should integrate SSR markers [53] or GWAS to map the quantitative trait loci (QTL) underlying these principal components, validating whether the D-value is a reliable predictor of genetic merit across environments [54]. In conclusion, this study demonstrates that maize landraces harbor complex trait architectures that balance reproductive output with resource allocation. By deconvoluting these traits into independent principal components, we provide breeders with not just a list of elite materials, but a strategic blueprint for optimizing yield potential through specific morphological adjustments.

## Conclusion

In summary, this study demonstrates that the 119 maize landraces evaluated herein harbor substantial phenotypic variability and high genetic diversity, confirming their status as vital reservoirs of adaptive alleles. Beyond merely quantifying diversity, our multivariate analysis elucidated the intrinsic trade-offs among key agronomic traits. The principal component analysis clarified that the divergence among these landraces is primarily structured along axes representing the balance between tassel proliferation and yield sink capacity, as well as grain-filling efficiency. This finding provides a biological explanation for the negative correlations observed between certain yield components and tassel traits, highlighting that optimizing resource allocation is crucial for yield improvement.

The identification of elite germplasm (e.g., Z102, Z7, Z89) through comprehensive scoring offers immediate practical value. These materials, characterized by favorable trait combinations identified via PCA, serve as ideal parental candidates for doubled haploid (DH) breeding programs [55] aimed at enhancing both architectural efficiency and productivity. Furthermore, the high heritability of the traits driving these principal components suggests that the phenotypic patterns observed here can be translated into genetic gains through marker-assisted selection.

Future perspectives should focus on bridging the gap between phenotype and genotype. While this study provides a robust framework for phenotypic evaluation, subsequent efforts should employ high-density molecular markers to validate the quantitative trait loci (QTL) underlying these key principal components. Integrating genomic selection models with the diverse genetic backgrounds identified in this study will facilitate the precise introgression of desirable alleles into elite cultivars, ultimately contributing to the development of maize varieties that are both high-yielding and resilient to environmental stresses.

## Supporting information

S1 Table The germplasm names of 119 maize landraces. (DOC)

S2 File Dataset of phenotypic quantitative traits. (XLS)

S3 Table P value of Pearson correlation analysis among 9 personality traits. Note: * represents significant correlation at P < 0.05; **represents significant correlation at P < 0.01.
(DOC)

S4 File Data from principal component analysis and comprehensive score analysis of 119 maize landraces. (XLS)

S5 File Dataset with 3 clusters. (XLS)
