# Supplementary material for: Phenotypic variation and diversity assessment in maize landraces
Source: PLoS One. 2026 Aug 14;21(8):e0355741. doi: 10.1371/journal.pone.0355741 (PMC13475892; doi:10.1371/journal.pone.0355741)
Supplement: Supplementary file 1 [file pone.0355741.s001.doc]

| Code | Germplasm name | Code | Germplasm name | Code | Germplasm name | Code | Germplasm name |
| --- | --- | --- | --- | --- | --- | --- | --- |
| Z1 | HuangQike | Z31 | KaoShanhuang | Z61 | ZaoShu | Z91 | HeiRuan |
| Z2 | DaHonggang | Z32 | HuangHuo | Z62 | ShuangJi | Z92 | BaiYuanli |
| Z3 | XiaoLihong_short | Z33 | DaLihong | Z63 | XiaoYang | Z93 | CaoYumi |
| Z4 | XiangYabai_1 | Z34 | 60cao | Z64 | 80RiHuang | Z94 | DaHuang |
| Z5 | ErPei | Z35 | 60bai | Z65 | TuBai | Z95 | HuangLi |
| Z6 | JiuChengDuosui | Z36 | GanZihuang | Z66 | HongYazizui | Z96 | BaiHuo |
| Z7 | LaoBaimaize | Z37 | JDDalihuang | Z67 | LocalBaidamaya | Z97 | CangZhouhuang |
| Z8 | ShuangSuibai | Z38 | XiaoMaya | Z68 | GaoShanbai | Z98 | CaoYumi |
| Z9 | BaiToushuang | Z39 | XiaoBai | Z69 | BaiMaya | Z99 | BaDahuang |
| Z10 | 66TOU | Z40 | WuLongzao | Z70 | FuBobai | Z100 | N0199 |
| Z11 | WanJinqueen | Z41 | DongGuangbai | Z71 | LiuShiri | Z101 | N0201 |
| Z12 | BaiXiaoriyue | Z42 | DengLonghong | Z72 | DaYumi | Z102 | N0203 |
| Z13 | 60_HaiJin | Z43 | ZhongShubai | Z73 | WuShe | Z103 | N0205 |
| Z14 | SiYangqiuzi | Z44 | AnDongmayaxuan | Z74 | N0147 | Z104 | JinHuanghou |
| Z15 | ErHuangcao | Z45 | HongRang | Z75 | NanHeduosui | Z105 | HSduosuibai |
| Z16 | DaJingzi | Z46 | BaiNuo | Z76 | TaKengbai | Z106 | Elinbai |
| Z17 | N0033 | Z47 | HuangQi | Z77 | BaiHuicha | Z107 | BaiMaya |
| Z18 | HuoLuHuang | Z48 | BaHangbai | Z78 | ErHuanghou | Z108 | 80Tianbai |
| Z19 | ErBenzi | Z49 | YiBaomian | Z79 | SanLicun | Z109 | 60RiCao |
| Z20 | N0039 | Z50 | BaiKezi | Z80 | WanBai | Z110 | DaPigu |
| Z21 | HuoBangzi | Z51 | DaHuangbang | Z81 | JiXianduosui | Z111 | HuaLaobaolian |
| Z22 | YuZibai | Z52 | LiuLiyan | Z82 | TPADmaya11 | Z112 | ZhenZhu |
| Z23 | DaLibai | Z53 | XiaoLihong_high | Z83 | JinLizi | Z113 | ChangSuiqunti |
| Z24 | XiangYabai_2 | Z54 | 60TianCao | Z84 | DaHuanggai | Z114 | BaoLiu |
| Z25 | HongZhoutu | Z55 | DaHongpao | Z85 | TieYuhong | Z115 | ChaoXian |
| Z26 | BaiSanlicun | Z56 | XiaoBaiLI | Z86 | WGBaimaya | Z116 | GouShi |
| Z27 | LaoBaiwan | Z57 | WuHua | Z87 | SanQiuhuang | Z117 | 22H2028 |
| Z28 | HuangErshucao | Z58 | HuangMaya | Z88 | 22H2042 | Z118 | 22h2116 |
| Z29 | XiaoJinzi | Z59 | BaiYuzi | Z89 | XiaoHuangyujiao | Z119 | 22H2124 |
| Z30 | GaoChengbai | Z60 | LiaoDongbai | Z90 | BaDahuang |  |  |
