# Supplementary material for: Phenotypic variation and diversity assessment in maize landraces
Source: PLoS One. 2026 Aug 14;21(8):e0355741. doi: 10.1371/journal.pone.0355741 (PMC13475892; doi:10.1371/journal.pone.0355741)
Supplement: Supplementary file 2 [file pone.0355741.s003.doc]

| TRAIT |  | SRN | GPR | TGW | TSL | TPAL | TSD | TBN | TSBN | AFBMA |
| --- | --- | --- | --- | --- | --- | --- | --- | --- | --- | --- |
| SRN | p-value | - |  |  |  |  |  |  |  |  |
| GPR | p-value | 0.00 | - |  |  |  |  |  |  |  |
| TGW | p-value | 0.00 | 0.00 | - |  |  |  |  |  |  |
| TSL | p-value | 0.51 | 0.00 | 0.00 | - |  |  |  |  |  |
| TPAL | p-value | 0.57 | 0.00 | 0.01 | 0.31 | - |  |  |  |  |
| TSD | p-value | 0.00 | 0.00 | 0.05 | 0.74 | 0.00 | - |  |  |  |
| TBN | p-value | 0.91 | 0.21 | 0.13 | 0.00 | 0.03 | 0.00 | - |  |  |
| TSBN | p-value | 0.66 | 0.01 | 0.00 | 0.45 | 0.00 | 0.00 | 0.00 | - |  |
| AFBMA | p-value | 0.04 | 0.81 | 0.38 | 0.09 | 0.45 | 0.27 | 0.00 | 0.88 | - |
|  | | | | | | | | | | |
